# Effect of Kangaroo Mother Care on the Psychological Stress Response and Sleep Quality of Mothers With Premature Infants in the Neonatal Intensive Care Unit

**DOI:** 10.3389/fped.2022.879956

**Published:** 2022-07-22

**Authors:** Wei-yan Chen, Ying-ying Wu, Meng-yan Xu, Tao-Hsin Tung

**Affiliations:** ^1^Nursing Department, Taizhou Hospital of Zhejiang Province Affiliated to Wenzhou Medical University, Linhai, China; ^2^Nursing Department, Hangzhou Women's Hospital (Hangzhou Maternity and Child Health Care Hospital), Hangzhou, China; ^3^Evidence-Based Medicine Center, Taizhou Hospital of Zhejiang Province Affiliated to Wenzhou Medical University, Linhai, China

**Keywords:** mothers of preterm infants, Kangaroo mother care, psychological stress, sleep quality, mother-infant separation

## Abstract

**Objective:**

To investigate the effect of kangaroo mother care on the psychological stress response and sleep quality of mothers with premature infants admitted to the neonatal intensive care unit (NICU).

**Methods:**

A randomized controlled design was used to recruit participants. The study recruited 126 mothers of premature infants in the NICU from January 2019 to January 2020. The participants were divided into the experimental and control groups according to the random number table method (63 mothers per group). The control group was managed with conventional premature infant treatment, nursing programme, and discharge education, whereas the experimental group was managed with a 4-week kangaroo mother care intervention. The psychological stress state and sleep status of mothers with preterm infants in both the groups were evaluated using the Symptom Check List 90 (SCL-90) and Assens Insomnia Scale (AIS).

**Results:**

After the intervention, the total SCL-90 score and factor scores such as coercion, interpersonal relationships, depression, anxiety, hostility and additional factors, were lower in the experimental group than those in the control group (*P* < 0.05). The total AIS score and the items such as night waking, total sleep time, total sleep quality, daytime mood and daytime body function were lower in the experimental group than those in the control group (*P* < 0.05).

**Conclusions:**

The Kangaroo mother care approach can relieve adverse psychological stress and improve the sleep status of mothers of NICU premature infants after mother-infant separation. It can be promoted and used in mothers of premature infants in the NICU to enhance physical and mental health.

## Introduction

Mother-infant separation due to admission of premature infants to the neonatal intensive care unit (NICU) is a stressor for postpartum women, affecting their physical and mental health. Studies have shown that 20% of mothers with premature infants in the NICU have varying degrees of depressive symptoms: 43% have moderate to severe anxiety (1) and 15% have both anxiety and depressive symptoms (2). Lee et al. (3, 4) reported that, in their study there were 80% of the mothers with premature infants had varying degrees of sleep problems after delivery, and approximately 66% of mothers with premature infants had a severely impaired sleep status. Therefore, it is an urgent task to find effective nursing measures to alleviate the adverse psychological stress and sleep status of mothers with premature infants after mother-infant separation. Kangaroo Mother Care (KMC), also known as skin-to-skin care, refers to placing the newborn upright on the mother's (or father's) chest for the desired warmth and security, named for the way marsupials such as kangaroos take care of their babies and toddlers. A large number of studies have confirmed that KMC can not only stabilize the vital signs of premature infants, promote growth and development (5), but also can reduce their hospitalization cycle and mortality (6). Previous studies have found that the KMC can play a certain role in regulating depression, irritability, and other negative emotions in postpartum mothers (7), it can be used not only to improve the maternal attachments in mothers with premature infants (8), but also reduce the maternal anxiety and stress status after premature birth (9). But, there was a study found that a prolonged periods KMC was an exhausting experience for mothers which can cause them lack of sleep and feel tired (10). However, at present, there are few published studies on the effects of KMC on the psychological stress response and sleep status of mothers with premature infants during mother-infant separation. The purpose of this study was to explore the effects of KMC on the psychological stress response and sleep quality of mothers with preterm infants after mother-infant separation and to provide a reference to improve the quality of life for these mothers.

## Methods

### Study Design and Participants

Mothers with premature infants were recruited from the NICU of Hangzhou Women's hospital from January 2019 to January 2020 as the subjects of our study continuously. The survival rate and hospitalization period of preterm infants are closely related to the gestational age of preterm birth. The preterm infants who were born too early (<28 weeks) or too late (more than 34 weeks) may conflict with the study cycle of this study. So in our study, the inclusion criteria were as follows: (1) mothers who gave birth to premature infants at 28–34 weeks of gestation, with whose baby had an anticipated stay at this NICU for at least 4 weeks following recruitment, (2) hospitalized premature infants with stable vital signs, (3) those not requiring a invasive ventilator for breathing, (4) those who were able to tolerate feeding, (5) mothers who were healthy and free from infectious and skin diseases, (6) mothers with the ability to read and comprehend language, (7) and those who provided informed and voluntary consent. The exclusion criteria included the following: (1) premature infants with severe organ disease, severe deformity, severe neonatal hypoxic-ischemic encephalopathy, and invasive ventilator therapy; (2) mothers of premature infants who were unable to participate in the study throughout; and (3) mothers with a history of major mental illness and severe obstetric complications. Those who withdrew from the research process, had incomplete data or information, who could not complete the 4-week intervention, and who had medical disputes, faced forced discharge, abandonment, or were transferred to hospitals for treatment were eliminated from the study. This study was approved by the Ethics Committee of Hangzhou Women's hospital.

### Randomization and Blinding

This study was a single-center, prospective, observational, pilot randomized controlled study. Randomization was 1:1 allocation. The participants were randomized to experimental or control group using a random number table prepared by an independent statistician. Random assignments were generated by an investigator who was not involved in the testing or training. Due to the nature of the intervention, it was difficult to use blinding procedure.

### Study Protocol

The subjects who were recruited but excluded from randomized distribution were given usual neonatal care. The control group was managed with routine treatment and a nursing programme for premature infants, all treatment and care were performed in the neonatal care unit, whereas the experimental group managed with KMC intervention based on the control group. The KMC intervention protocol followed with: the doctor determined the time of the first KMC implementation according to the condition of the premature infant. The NICU has a separate room where kangaroo cares can performed, the same room the each time. The KMC was set to be performed between 9:00 and 12:00 every morning, 1 h each time, three times a week, for four consecutive weeks. During KMC, researchers provided effective guidance and communication to mothers with premature infants, instructed on effective breastfeeding, and provided good breastfeeding-related education when KMC was performed for the first time; the mothers were taught the general basic care and parenting knowledge required to handle newborns. The doctors answered the questions to the mothers patiently and transmitted relevant information during their duration at the hospital effectively. For mothers who had higher SCL-90 scale scores, targeted psychological care was provided: listen and comprehend to the mothers' feelings and needs, Mainly aiming at the doubts and worries from mothers which researchers should be explaining well, giving some comfort and answering the questions timely.

### Evaluation Tool

#### General Information Questionnaire

The questionnaire was initially designed by the researchers, and revised and improved to determine the final content after consulting clinical nursing experts. It included age, education level, current residence, monthly household income, gestational age, number of total pregnancies, mode of delivery, whether the first child, birth weight of the premature infant, payment method for medical expenses, etc. The general information questionnaire are shown in Table 1.

**Table 1:** General information questionnaire.

No.___________ name.____________ date._____________	
**In order to know the general situation of your childbirth, please tick “**✓**” in the corresponding selection, thank you for your cooperation**	
Age	____th
Whether the Han nationality	Yes□No□
Education level	Primary school and below□ Junior middle school□ Senior middle school□ College□ Undergraduate and above□
Current residence	City□ Non city□
Monthly household income	Below 5,000□ 5,000–10,000□ More than 10,000□
Mode of Delivery	Natural delivery□ Cesarean section□
Gestational age of the fetus	____week
Fetal weight	____kg
Whether first child	Yes□ No□
Payment for medical expenses	Medical insurance□ at own expense□

#### SCL-90

The SCL-90 (11) was compiled by Derogatis in 1975. It is one of the most widely used mental health assessment scales for adults aged >16 years. The scale comprises 90 items using a 5-point scoring method (from 1 to 5), with a total score of 90–450. The higher the score, the more serious the psychological condition, which reflects the psychological symptoms of 10 aspects: somatization, coercion, interpersonal relationships, anxiety, depression, hostility, terror, paranoia, psychosis, and additional factors. Cronbach's alpha coefficient of the scale was 0.974, and the test-retest reliability was 0.962.

#### AIS

The AIS is an internationally recognized self-measurement scale for sleep quality. The scale includes eight items: sleep latency period (time taken to fall asleep after the lights are turned off), night waking, earlier waking than expected, total sleep quality (regardless of the length of sleep), total sleep time, daytime body function (physical or mental: e.g. memory, cognition, and attention), daytime mood, and daytime fatigue. Each item is based on a 4-Level scoring method (from 0 to 3 points) and a total score of 0 to 24 points. A total score of 0–3 points indicates no sleeping disorder, 4–5 points indicates a possible sleeping disorder, and ≥6 points indicates a sleeping disorder. The higher the score, the worse the sleep quality. The scale's internal Consistency (Cronbach's alpha) coefficient was 0.90 and its test-retest reliability was 0.92 (12).

### Calculation of Sample Size

According to the pre-experimental results, the total score of SCL-90 was the primary outcome index of the study, and the sample size was estimated using the formula *N*1 = *N*2 = 2 × [(μα + μβ)σ/δ]^2^ for the comparison of the mean of the two samples (13). Take α = 0.05, β = 0.10, two-sided test, according to the pre-experiment results, the total score difference of SCL-90 between the two groups was 19, and standard deviation (σ) was 23.26, the required sample size for each group is calculated *N*1 = *N*2 ≈ 55, considering possible loss to follow-up, the sample size was expanded by 20%, and finally 66 cases were determined in each group.

### Data Collection

All data in this study were collected by researchers who have worked for more than 10 years and have solid professional knowledge and clinical experience. They have undergone an unified training before the start of the study and are proficient in the application and intervention methods of KMC. Before the intervention, mothers in both of the groups were asked to complete the general information questionnaire, SCL-90 scale, and AIS scale to evaluate their psychological stress state and sleep status prior to the intervention. After 4 weeks, the SCL-90 and AIS scales were completed again to evaluate the psychological stress state and sleep status after the intervention. In order to ensure the completeness and accuracy of filling with the information, all participants were conducted in a same room for a quiet and comfortable environment, and the researchers were not allowed to give suggestive language prompts, and all questionnaires were distributed and recovered on the spot.

### Statistical Analysis

SPSS 21.0 medical statistics software was used for statistical analysis, enumeration data were described by the number of cases [*n* (%)], statistical inference between groups was performed by chi-square or Fisher's exact tests, measurement data were expressed as mean ± standard deviation (*x* ± s), the independent samples *t*-test was used for comparison between groups, and the paired samples t-test was used for comparison within groups. The analysis of covariance (ANCOVA) performed to clarify the results of the SCL and AIS scores between the experiment and control groups after the KMC intervention. Statistical significance was set at *P* < 0.05.

## Results

**Figure 1:**
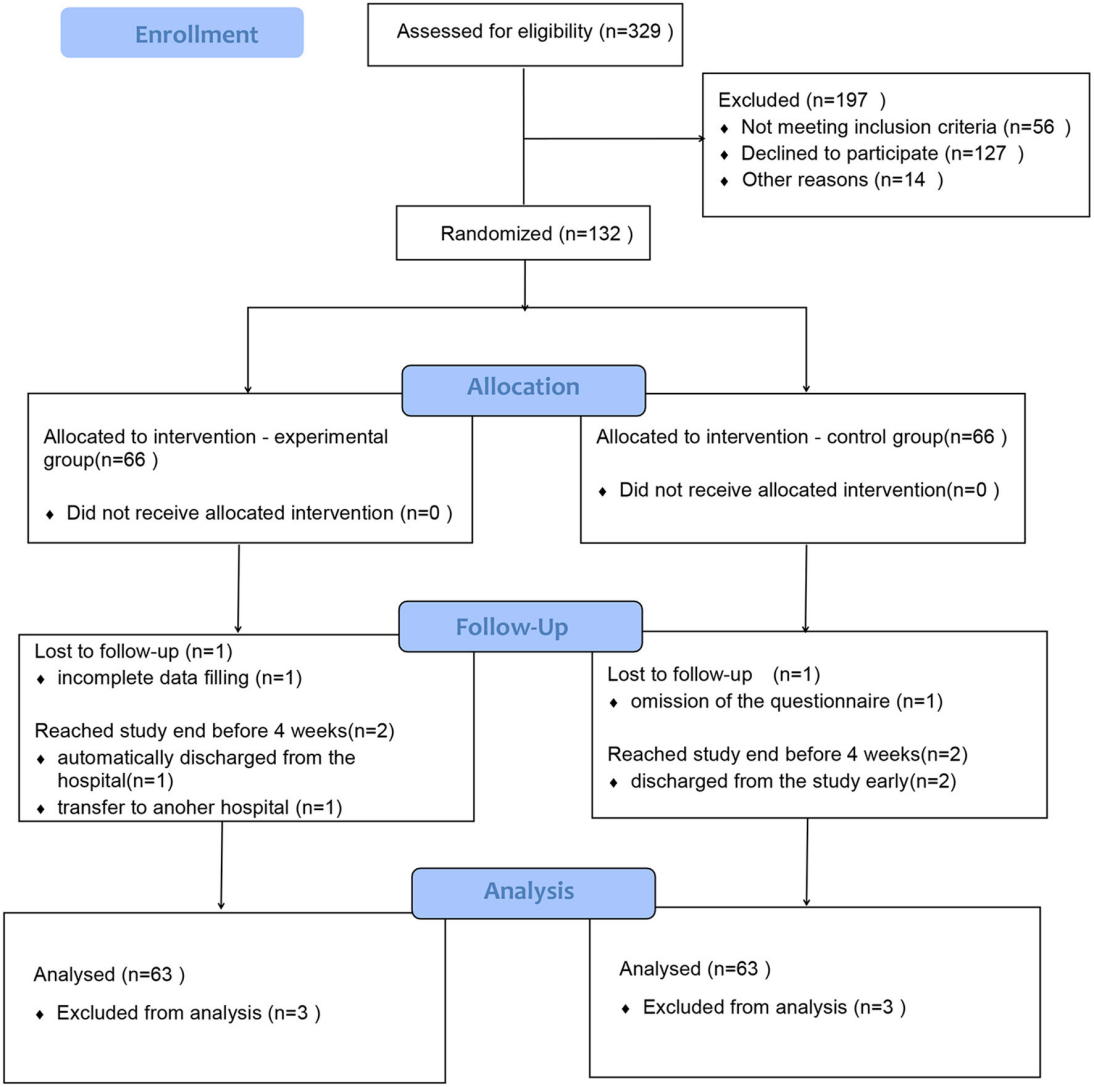
Participant disposition by allocation to experimental or control group.

The first participant was enrolled on January 6th, 2019, and the follow-up was completed on January 10th, 2020. Eligibility was assessed in 329 patients, and 132 patients were enrolled and randomized. All subjects were mothers of premature infants admitted to the NICU of Hangzhou womwen's Hospital, with 66 cases in each group. In the experimental group, one withdrew from the study due to transfer to another hospital, one was automatically discharged from the hospital and did not meet the study requirements, and one was excluded due to incomplete data filling. In the control group, two were discharged from the study early, and one was excluded from the study due to omission of the questionnaire. For those who were excluded were not analyzed in our study, so at last, there were 63 subjects analyzed in each group. The flowchart of participant inclusion and exclusion is shown in Figure 1.

### Comparison of General Characteristics Between the Two Groups

There was no significant difference in the general characteristics between the experimental and control groups. The results are shown in Table 2.

**Table 2:** Comparison of general characteristics between the experimental and control groups [*x* ± *s, n* (%)].

**Variable**	**Category**	**Experimental group (*n* = 63)**	**Control group (*n* = 63)**	**Statistics**	***P*-Value**
				**χ^2^/*t***	
Mothers' age (y, $\bar{x}$ ±*s*)		30.38 ± 3.49	30.11 ± 4.06	−0.400^#^	0.690
Education level (*n*, %)	Below junior high school	0 (0.00)	0 (0.00)	1.598*	0.450
	High school, secondary school	6 (9.52)	10 (15.87)		
	College	17 (26.98)	19 (30.16)		
	Undergraduate and above	40 (63.49)	34 (53.97)		
Current residence (*n*, %)	Urban	54 (85.71)	50 (79.37)	0.881*	0.348
	Non-urban	9 (14.29)	13 (20.63)		
Monthly household income (*n*, %)	<5,000	0 (0.00)	0 (0.00)	0.403*	0.525
	5,000–10,000	13 (20.63)	16 (25.40)		
	>10,000	50 (79.37)	47 (74.60)		
Gestational week (*n*, %)	28–30	6 (9.52)	2 (3.17)	2.322*	0.313
	30–32	13 (20.63)	16 (25.40)		
	32–34	44 (69.84)	45 (71.43)		
Birth weight of the premature infant (*n*, %)	<1 kg	0 (0)	1 (1.59)	–^Δ^	0.545
	1–1.5 kg	8 (12.70)	5 (7.94)		
	1.5–2.0 kg	32 (50.79)	28 (44.44)		
	2.0–2.5 kg	18 (28.57)	25 (39.68)		
	>2.5 kg	5 (7.94)	4 (6.35)		
Mode of delivery (*n*, %)	Natural delivery	23 (36.51)	26 (41.27)	0.301*	0.584
	Cesarean section	40 (63.49)	37 (58.73)		
Pregnancy times (*n*, %)	First born	51 (80.95)	45 (71.43)	1.575*	0.209
	Not the first child	12 (19.05)	18 (28.57)		
Whether twins (*n*, %)	Yes	7 (11.11)	9 (14.29)	0.286*	0.593
	No	56 (88.88)	54 (85.71)		
Payment method for medical expenses (*n*, %)	Medical insurance	63 (100.00)	63 (100.00)	–	–
	Non-medical insurance	0 (0)	0 (0)		

The * symbol indicates Chi-square test;

*The # symbol indicates t-test;*

*The Δ symbol indicates Fisher's exact test*.

### Comparison of SCL-90 Scale Scores Between the Two Groups Before and After the Intervention

Our results contain a large number of comparisons, so we adjust *P*-value to avoid type 1 errors. Statistical significance in scl-90 was set at *P* < 0.05/11 (*P* < 0.0045). Before the intervention, there was no significant difference in the SCL-90 factors and total score between the experimental and control groups. After the intervention, in experiment group, the total score and factors such as somatization, coercion, interpersonal relationships, depression, anxiety, hostility (*P* < 0.001) and additional factor (*P* = 0.001) were decreased significantly compared with those before the intervention. While in the control group only somatization (*P* < 0.001) decreased significantly. After adjustment for the pre-tests, the total score (127.85 ± 19.99 vs. 147.31 ± 18.76, *P* < 0.001), coercion (1.63 ± 0.45 vs. 1.78 ± 0.30, *P* < 0.001), interpersonal relationship (1.18 ± 0.20 vs. 1.89 ± 0.39, *P* < 0.001), depression (1.53 ± 0.40 vs. 1.92 ± 0.40, *P* < 0.001), anxiety (1.16 ± 0.17 vs. 1.64 ± 0.38, *P* < 0.001) and hostility (1.59 ± 0.35 vs. 1.73 ± 0.35, *P* = 0.001) were statistically significant difference after the intervention between experiment and control groups (Table 3).

**Table 3:** Comparison of each factor score and total score of SCL-90 scale between the experimental and control groups before and after the intervention (x ± s).

**Factors**	**Experimental group (*****n*** **=** **63)**		**Control group (*****n*** **=** **63)**		**t_**1**_**	**P_**1**_**	**t_**2**_**	**P_**2**_**	**t_**3**_**	**P_**3**_**	**t_**4**_**	**P_**4**_**	**F**	**P_**5**_**
	**Before**	**After**	**Before**	**After**										
Somatization	1.51 ± 0.47	1.37 ± 0.34	1.60 ± 0.33	1.47 ± 0.26	−1.283	0.202	5.741	<0.001	8.470	<0.001	−1.846	0.067	3.513	0.063
Coercion	1.76 ± 0.57	1.63 ± 0.45	1.80 ± 0.34	1.78 ± 0.30	−0.453	0.651	5.001	<0.001	0.953	0.344	−2.152	0.034	27.501	<0.001
Interpersonal relationship	1.79 ± 0.59	1.18 ± 0.20	1.86 ± 0.43	1.89 ± 0.39	−0.775	0.440	9.522	<0.001	−0.766	0.447	−12.813	<0.001	247.293	<0.001
Depression	1.93 ± 0.70	1.53 ± 0.40	2.01 ± 0.34	1.92 ± 0.40	−0.813	0.418	9.111	<0.001	2.001	0.051	−5.470	<0.001	58.775	<0.001
Anxiety	1.72 ± 0.68	1.16 ± 0.17	1.68 ± 0.39	1.64 ± 0.38	0.323	0.747	7.085	<0.001	1.163	0.249	−9.347	<0.001	118.282	<0.001
Hostility	1.78 ± 0.54	1.59 ± 0.35	1.83 ± 0.42	1.73 ± 0.35	−0.523	0.602	6.209	<0.001	2.717	0.009	−2.234	0.027	10.676	0.001
Terror	1.35 ± 0.39	1.28 ± 0.36	1.27 ± 0.22	1.23 ± 0.23	1.405	0.163	1.667	0.100	1.898	0.062	1.062	0.290	0.154	0.696
Paranoia	1.63 ± 0.58	1.54 ± 0.54	1.67 ± 0.43	1.55 ± 0.41	−0.486	0.628	2.001	0.051	1.951	0.056	−0.126	0.900	0.064	0.801
Psychosis	1.39 ± 0.36	1.34 ± 0.28	1.32 ± 0.28	1.25 ± 0.27	1.316	0.191	1.944	0.056	1.859	0.068	1.838	0.068	1.678	0.198
Additional	1.82 ± 0.56	1.70 ± 0.42	1.83 ± 0.40	1.83 ± 0.33	−0.097	0.923	3.573	0.001	−0.016	0.987	−2.024	0.045	7.692	0.006
Total score	150.61 ± 38.80	127.85 ± 19.99	152.75 ± 23.69	147.31 ± 18.76	−0.373	0.710	8.824	<0.001	1.429	0.156	−6.505	<0.001	150.014	<0.001

*t_1_ and P_1_ are the comparisons of the two groups before the intervention; t_2_ and P_2_ are the comparisons of the experimental group before and after the intervention; t_3_ and P_3_ are the comparisons of the control group before and after the intervention; t_4_ and P_4_ are the comparisons of the two groups after the intervention. F and P_5_ are the analysis of covariance (ANCOVA) comparisons of the two groups after the intervention*.

### Comparison of AIS Scale Scores Between the Two Groups Before and After the Intervention

Our results contain a large number of comparisons, so we adjust P-value to avoid type 1 errors. Statistical significance in AIS was set at P < 0.05/9 (P < 0.0056). Before the intervention, there was no significant difference between the two groups of AIS items and total score. After the intervention, in experiment group, the total score, earlier waking, total sleep time, total sleep quality, daytime mood, daytime body function (P < 0.001) and night waking (P < 0.05) were decreased significantly compared with those before the intervention. While in the control group, there was no significant difference compared with those before and after. After adjustment for the pre-tests, the total score (6.41 ± 2.6 vs. 8.98 ± 2.34, P < 0.001), total sleep quality (0.86 ± 0.74 vs. 1.51 ± 0.59, P < 0.001), daytime mood (0.51 ± 0.59 vs. 1.06 ± 0.59, P < 0.001), daytime body function (0.52 ± 0.56 vs. 1.00 ± 0.54, P < 0.001) were statistically significant difference after the intervention between experiment and control groups (Table 4).

**Table 4:** Comparison of each item score and total score of the AIS scale between the experimental and control groups before and after the intervention (x ± s).

**Factors**	**Experimental group (n** **=** **63)**		**Control group (n** **=63)**		**t_**1**_**	**P_**1**_**	**t_**2**_**	**P_**2**_**	**t_**3**_**	**P_**3**_**	**t_**4**_**	**P_**4**_**	**F**	**P_**5**_**
	**Before**	**After**	**Before**	**After**										
Sleep latency	1.03 ± 0.95	1.08 ± 0.75	1.02 ± 0.85	1.03 ± 0.69	0.099	0.921	−0.375	0.709	−0.178	0.859	0.370	0.712	0.132	0.717
Night waking	1.10 ± 0.88	0.84 ± 0.65	1.18 ± 0.68	1.16 ± 0.70	−0.567	0.572	2.901	0.005	0.163	0.871	−2.632	0.010	7.298	0.008
Earlier waking	1.37 ± 0.58	0.91 ± 0.61	1.25 ± 0.47	1.08 ± 0.27	1.182	0.240	5.283	<0.001	2.640	0.010	−2.061	0.042	3.321	0.071
Total sleep time	1.22 ± 0.73	0.87 ± 0.55	1.30 ± 0.71	1.13 ± 0.58	−0.619	0.537	3.839	<0.001	2.377	0.021	−2.511	0.013	6.392	0.013
Total sleep quality	1.43 ± 0.91	0.86 ± 0.74	1.56 ± 0.76	1.51 ± 0.59	−0.851	0.396	4.713	<0.001	0.504	0.616	−5.462	<0.001	30.128	<0.001
Daytime mood	1.06 ± 0.91	0.51 ± 0.59	1.13 ± 0.63	1.06 ± 0.59	−0.453	0.651	5.261	<0.001	0.629	0.531	−5.265	<0.001	28.914	<0.001
Daytime body function	1.13 ± 0.83	0.52 ± 0.56	1.06 ± 0.78	1.00 ± 0.54	0.442	0.660	5.615	<0.001	0.683	0.497	−4.846	<0.001	28.476	<0.001
Daytime sleepiness	0.92 ± 0.45	0.83 ± 0.52	1.06 ± 0.50	1.02 ± 0.55	−1.677	0.096	1.426	0.159	0.725	0.471	−1.982	0.049	1.772	0.186
Total score	9.25 ± 4.35	6.41 ± 2.69	9.56 ± 3.65	8.98 ± 2.34	−0.422	0.674	6.340	<0.001	1.879	0.065	−5.724	<0.001	41.674	<0.001

*Note: t_1_ and P_1_ are the comparisons of the two groups before the intervention; t_2_ and P_2_ are the comparisons of the experimental group before and after the intervention; t_3_ and P_3_ are the comparisons of the control group before and after the intervention; t_4_ and P_4_ are the comparisons of the two groups after the intervention. F and P_5_ are the analysis of covariance (ANCOVA) comparisons of the two groups after the intervention*.

## Discussion

### Interaction Between Adverse Psychological Stress Response and Sleep Status of Mothers With Premature Infants

Psychological stress is a process in which individuals perceive the threat of stressors through cognition and evaluation under certain environmental stimuli, causing changes in psychological and physiological functions (14). When premature birth occurs, a lack of awareness of this stress will increase the psychological pressure on mothers and they are prone to adverse psychological stress reactions such as anxiety and depression. If these pressures are not resolved in time, sleep problems occur to varying degrees, which affect the mother's postpartum recovery and future family care. Sleep quality and psychological state are complementary to each other (15). If the mother does not receive adequate rest, she will not have enough energy to face daily self- adjustment and breastfeeding of the baby, which will worsen her psychological state and further disturb her sleep (16). Poor sleep can also lead to endocrine disorders in the body, causing greater emotional fluctuations, which will, again, affect sleep, thus forming a vicious cycle (17).

### KMC Alleviates the Psychological Stress of Mothers During Hospitalization

Mothers often doubt their own abilities as mothers when their premature infants are admitted to the NICU, and attribute the responsibility of mother-infant separation to themselves, which is easy to induce an anxiety and depression from separation (18). In our study, through the KMC, continuous communication and intimate contact from skin-to-skin between mothers and their babies are possible, researchers encourage the mothers to communicate with eyes and words with their premature infants, and provide effective breastfeeding guidance and education to the mothers of premature, and solve the problems in feeding process in a timely and targeted manner. This feeling is very real when mothers are able to hug and touch their children, it not only can reduces maternal stress, improves negative emotions in mothers (eg., anxiety and depression), but also promotes positive parent-child relationships (9, 19). The result of our study showed that after the intervention, the total score, anxiety and depression of experimental group were decreased significantly compared with those in the control group (*P* < 0.001). It indicate that KMC can promote breastfeeding and enhance the self-efficacy of mothers with premature infants. At the same time, it can reduce the psychological pressure, then relieve the anxiety, depression and other negative emotions caused by the separation of mother-infant. This is consistent with the study by Sinha et al. (7),which showed that early postpartum maternal depression and anxiety symptoms are negatively correlated with the initiation and duration of breastfeeding, the earlier the mothers started breastfeeding, the lower the levels of postpartum anxiety and depression and the better their psychological state.

At present, the vast majority of NICUs in our country implement with closed management based on factors such as infection prevention and insufficient staff.

Mothers have rarely opportunity to contact with their babies who were hospitalized before the discharge (20), such mother-infant separation is a great challenge for mothers of premature infants. Lack of relevant information about the premature infants in the NICU, the uncertainty of the disease limits the mother's psychological adjustment and affects the mother's physical and mental health (21). In this study, through the KMC, the researchers listen and comprehend to the mothers' feelings and needs, answer the relevant questions of the mothers patiently and convey all relevant information of the premature infants during their Hospitalization effectively. Previous studies have pointed out that good transmission is an important part of the mother's support, guiding the mother's adaptation into her new role, which can make her feel comfortable and trustworthy, eliminate self-blame and inferiority, improve the sensitivity of interpersonal relationships, and provide reassurance (22, 23). The results of the study showed that after the intervention, the experimental group was lower than the control group in items of interpersonal relationship, coercion and hostility. It shows that KMC can provide information support for mothers, while promoting role adaptation, it can not only relieve adverse stress reactions such as mothers' anxiety, depression and coercion, and can also improve the hostility and interpersonal relationship factors of premature mothers. This is consistent with the results of the study by Zhang et al. (24), which showed that KMC provides mothers with a means to learn more about their newborns in stressful situations, which enables them to generate strong positive emotions and reconcile their feelings about preterm birth, thereby indicating an emotional therapy.

### Effects of KMC on Sleep Quality of Mothers With Premature Infants in the NICU

The AIS is an internationally recognized self-assessment scale for assessing sleep quality. A total score of more than 6 points indicates the existence of sleep disorders, and the higher the score, the worse the condition of sleep (12). Due to the particularity of the NICU ward in our country, mothers are unable to visit their preterm infants who are hospitalized at all times. And anxiety, uncertainty and feelings of powerlessness can negatively impact sleep; Worry about the condition changes and prognosis of premature infants; Lack of postpartum breastfeeding, etc., these factors increase the psychological pressure of mothers, which in turn makes it difficult to fall asleep at night, and even wake up easily after falling asleep, and wake up earlier, which ultimately greatly reduces the quality of sleep. In our study, before the intervention, the total score of the mothers of AIS in each group was >6 points, it indicating that the mothers with premature infants have a certain degree of sleep disturbance. The results of our study are consistent with the findings of other studies (25), and the sleep status of the mothers with premature infants in the NICU was not optimistic. In this study, during the KMC intervention, researchers deliver good information of premature infants in NICU to their mothers to help them understanding information about their infants in NICU, which reduce the sense of disease uncertainty of the premature mothers; And at the KMC, the researchers provided professional and effective guidance to the mothers of premature infants, giving them the opportunity to learn and participate in the care of the premature infants, which enhanced the mothers' self-confidence and laid the foundation for the care of the premature infants after discharge. After the intervention, the results showed that the experimental group was significantly lower in the total score and items such as night waking, earlier waking, total sleep time, total sleep quality, daytime mood and daytime body function than those before the intervention. And it also scored lower in total score, total sleep quality, daytime mood and daytime body function than those in the control group. The results indicated that KMC can significantly improve the sleep status of mothers with premature Infants, improved their total sleep quality, thereby improving the mother's physical function and mood during the day. This conclusion is consistent with previous research results (26, 27), which showed that KMC can improve the sleep quality of mothers with premature infants during mother-infant separation and promote the physical and mental recovery of postpartum mothers. Combined with the analysis of this study, the improvement in sleep status in the experimental group may also be related to the fact that KMC can reduce the psychological pressure of mothers with premature infants, making them more relaxed, stabilize negative emotions, improve self-efficacy, and ultimately improve maternal sleep quality.

### Limitations

There were several limitations should be discussed in this study. Firstly, there was limitation in the selection of diverse backgrounds of research objects. Each mother with the premature infant is an individual, due to the different social experiences and backgrounds of individuals, mothers of preterm infants have different psychological endurance and stress responses in the face of preterm birth events. Secondly, our study population was limited to mothers with premature infants at 28–34 weeks of gestation, and the findings may not be applicable to mothers with other weeks of gestation premature infants. Thirdly, we only considered KMC practice for 1 h, for three times a week for 4 weeks, the establishment of this intervention may not good enough. Finally, this research is limited by space, time and manpower, only a NICU from Hangzhou women's hospital was selected as the research site, so, there were certain limitations in the determination and selection of the sample size. In future studies, multi- center, large samples can be conducted, with qualitative and quantitative studies combined, and extended to various primary hospitals and communities, and provide a more reliable basis for clinical application.

## Conclusions

In conclusion, the Kangaroo mother care as a non-pharmacological intervention can relieve adverse psychological stress and improve the sleep status of mothers of NICU premature infants after mother-infant separation. It can be promoted and used in mothers of premature infants in the NICU to enhance physical and mental health.

## Data Availability Statement

The original contributions presented in the study are included in the article/supplementary material, further inquiries can be directed to the corresponding authors.

## Ethics Statement

The studies involving human participants were reviewed and approved by Hangzhou Women's Hospital (Hangzhou Maternity and Child Health Care Hospital) Ethics Committee. The patients/participants provided their written informed consent to participate in this study.

## Author Contributions

Conception and design: W-yC and M-yX. Acquisition, analysis, or interpretation of data: W-yC, M-yX, and Y-yW. Statistical analysis and drafting of the manuscript: W-yC. Supervision: T-HT. All authors have read and agreed to the published version of the manuscript.

## Funding

This study was supported by the Zhejiang Enze Medical Center Group Research Project (No. 20EZD68) and the Natural Science Foundation of Zhejiang Province (No. LBY21H040002).

## Conflict of Interest

The authors declare that the research was conducted in the absence of any commercial or financial relationships that could be construed as a potential conflict of interest.

## Publisher's Note

All claims expressed in this article are solely those of the authors and do not necessarily represent those of their affiliated organizations, or those of the publisher, the editors and the reviewers. Any product that may be evaluated in this article, or claim that may be made by its manufacturer, is not guaranteed or endorsed by the publisher.
